# Enhanced attention-related alertness following right anterior insular cortex neurofeedback training

**DOI:** 10.1016/j.isci.2024.108915

**Published:** 2024-01-15

**Authors:** Jeanette Popovova, Reza Mazloum, Gianluca Macauda, Philipp Stämpfli, Patrik Vuilleumier, Sascha Frühholz, Frank Scharnowski, Vinod Menon, Lars Michels

**Affiliations:** 1 Department of Neuroradiology, University Hospital of Zurich, 8091 Zurich, Switzerland; 2 Neuroscience Center Zurich, University of Zurich and ETH Zurich, 8057 Zurich, Switzerland; 3 Department of Psychology, University of Zurich, 8050 Zurich, Switzerland; 4 Department of Health Sciences and Technology, ETH Zurich, 8092 Zurich, Switzerland; 5 MR-Center of the Department of Psychiatry, Psychotherapy and Psychosomatics and the Department of Child and Adolescent Psychiatry, Psychiatric Hospital, University of Zurich, 8032 Zurich, Switzerland; 6 Department of Neurosciences and Clinic of Neurology, Laboratory for Neurology and Imaging of Cognition, University of Geneva, 1211 Geneva, Switzerland; 7 Department of Psychology, University of Oslo, 0851 Oslo, Norway; 8 Department of Cognition, Emotion, and Methods in Psychology, Faculty of Psychology, University of Vienna, 1010 Vienna, Austria; 9 Department of Psychiatry & Behavioral Sciences, Stanford University School of Medicine, Stanford, CA, USA; 10 Department of Neurology & Neurological Sciences, Stanford University School of Medicine, Stanford, CA, USA; 11 Wu Tsai Neurosciences Institute, Stanford University School of Medicine, Stanford, CA, USA

**Keywords:** Biological sciences, Neuroscience, Sensory neuroscience, Techniques in neuroscience

## Abstract

The anterior insular cortex, a central node of the salience network, plays a critical role in cognitive control and attention. Here, we investigated the feasibility of enhancing attention using real-time fMRI neurofeedback training that targets the right anterior insular cortex (rAIC). 56 healthy adults underwent two neurofeedback training sessions. The experimental group received feedback from neural responses in the rAIC, while control groups received sham feedback from the primary visual cortex or no feedback. Cognitive functioning was evaluated before, immediately after, and three months post-training. Our results showed that only the rAIC neurofeedback group successfully increased activity in the rAIC. Furthermore, this group showed enhanced attention-related alertness up to three months after the training. Our findings provide evidence for the potential of rAIC neurofeedback as a viable approach for enhancing attention-related alertness, which could pave the way for non-invasive therapeutic strategies to address conditions characterized by attention deficits.

## Highlights

• The anterior insular cortex is critically involved in attentional functioning
• Healthy participants increased activity in their rAIC using rt-fMRI neurofeedback
• rAIC activity upregulation enhanced alertness during attention task
• Behavioral effects persisted up to three months after neurofeedback training

## Graphical abstract

**Figure:**
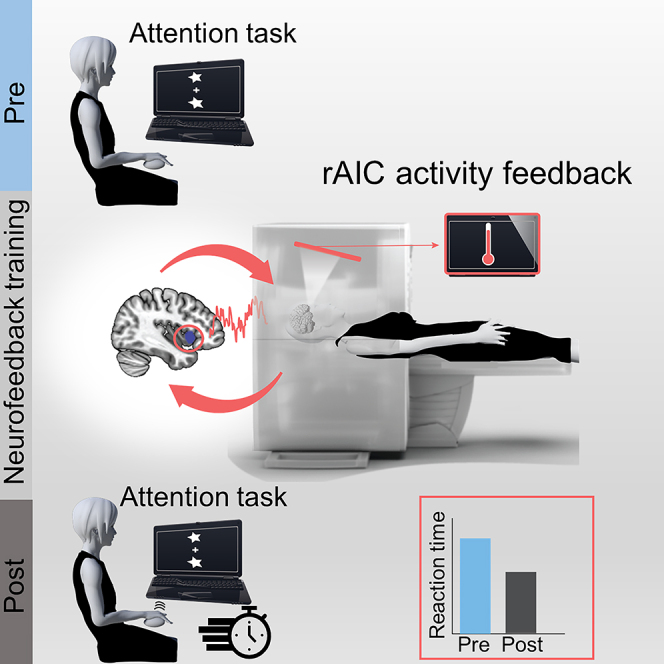

## Introduction

Cognitive control and attention facilitate flexible and goal-directed behaviors in an ever-changing environment. Deficits in these cognitive functions are debilitating for patients suffering from attention-deficit hyperactivity disorder (ADHD),^1^ schizophrenia,^2^ traumatic brain injury,^3^ Parkinson’s disease,^4^ and other psychiatric and neurological disorders. Converging evidence from neuroimaging studies highlights the pivotal role of the right anterior insular cortex (rAIC) as a key hub of the salience network^5^ for the implementation of cognitive control and attention.^6^^,^^7^^,^^8^^,^^9^^,^^10^ Despite this proposed role of the rAIC, little is known about the feasibility of improving cognition and attention by modulation of neural activity in the rAIC. Here, we used a longitudinal real-time fMRI (rt-fMRI) neurofeedback training design to modulate rAIC activity and investigate the impact on attention and cognition.

The salience network encompasses the bilateral anterior insula and anterior cingulate cortex (ACC) and facilitates the detection and filtering of salient events.^5^^,^^7^^,^^10^ An influential neurocognitive model has emphasized the critical role of the anterior insula by extending its function to the triggering of other large-scale brain networks and thereby influencing higher cognitive processes such as attention and cognitive control.^7^ For example, using analysis of dynamic interactions, it has been shown that the rAIC drives the switching from the deactivation of the default mode network (DMN) to activation of the central executive network during rest and attention tasks.^10^ In line with these findings, stable rAIC activity has been reported across a wide range of cognitive tasks suggesting a general role in cognitive control.^9^^,^^11^^,^^12^^,^^13^^,^^14^ Using multivariate dynamical systems state-space models, Cai et al.^12^ found that the causal influence from the rAIC to the ACC increased with higher demand for cognitive control and correlated with general cognitive control abilities across three different cognitive tasks. A study employing graph theory analysis of fMRI data found that the rAIC has strong connections to other important “inhibition nodes” during response inhibition, suggesting that the rAIC may function as a “choirmaster” orchestrating cognitive control.^15^ The proposed critical role of the rAIC in the implementation of conscious cognitive control is also highlighted in a study using a Go/NoGo task involving subliminal cues.^16^

Despite these advances, the causal role of the rAIC in cognitive control and attention is not known due to the cross-sectional (single time point) designs and correlational analyses. Crucially, it is not known whether modulation of rAIC activity has long-term effects on attention. To directly address this question we used rt-fMRI neurofeedback, a widely used technique in which individuals are trained to voluntarily increase or decrease their own neural activity from specific brain regions (e.g., studies by Scharnowski et al and Young et al.,^17^^,^^18^) and even entire networks (e.g., studies by Koush et al., Pamplona et al., and Ramot et al.,^19^^,^^20^^,^^21^).

Studies attempting to increase cognitive control in individuals with ADHD using rt-fMRI neurofeedback have typically targeted the dorsal ACC^22^ and right ventrolateral frontal cortex.^23^ In healthy participants, short-term beneficial effects on sustained attention have been reported using network-based rt-fMRI neurofeedback training where participants trained to increase the difference in activity between the DMN and a combination of brain regions of the frontoparietal and dorsal attention network.^20^ Furthermore, participants improved on a sustained attention task where they had to focus on either face or scene aspects of composite stimuli after they performed the same task during a rt-fMRI neurofeedback setup where the task difficulty was adapted based on their individual whole-brain multivariate brain signatures indicating whether they were focusing on the relevant category.^24^ While previous studies have demonstrated the effectiveness of rt-fMRI neurofeedback on attention by using feedback from multiple brain areas, our study uniquely targets the rAIC for this purpose. This approach marks an advancement in the specificity and focus of neurofeedback interventions.

Previous studies in healthy participants^25^^,^^26^^,^^27^^,^^28^ and psychiatric populations^29^^,^^30^ have already successfully shown the feasibility of anterior insula activity modulation through rt-fMRI neurofeedback training. These studies have focused on behavioral changes in emotion processing and have provided participants with explicit strategies involving emotional content to regulate the feedback signal. Providing participants with specific emotion regulation strategies can confound the interpretation of the resulting effects, as changes in brain activity and behavior following rt-fMRI may be caused by neural pattern changes related to the cognitive strategies rather than the feedback control itself.^31^^,^^32^ Furthermore, previous neurofeedback studies targeting the rAIC have not assessed changes in attention and only recorded short-term behavioral effects of self-regulation. In the present study, we address this gap in the literature and use rt-fMRI neurofeedback to specifically target the rAIC with multiple control conditions and assess both short- and long-term behavioral changes in attention. We sought to determine whether individuals can voluntarily modulate neural activity, and whether this modulation leads to sustained improvements in attention.

**Figure 1:**
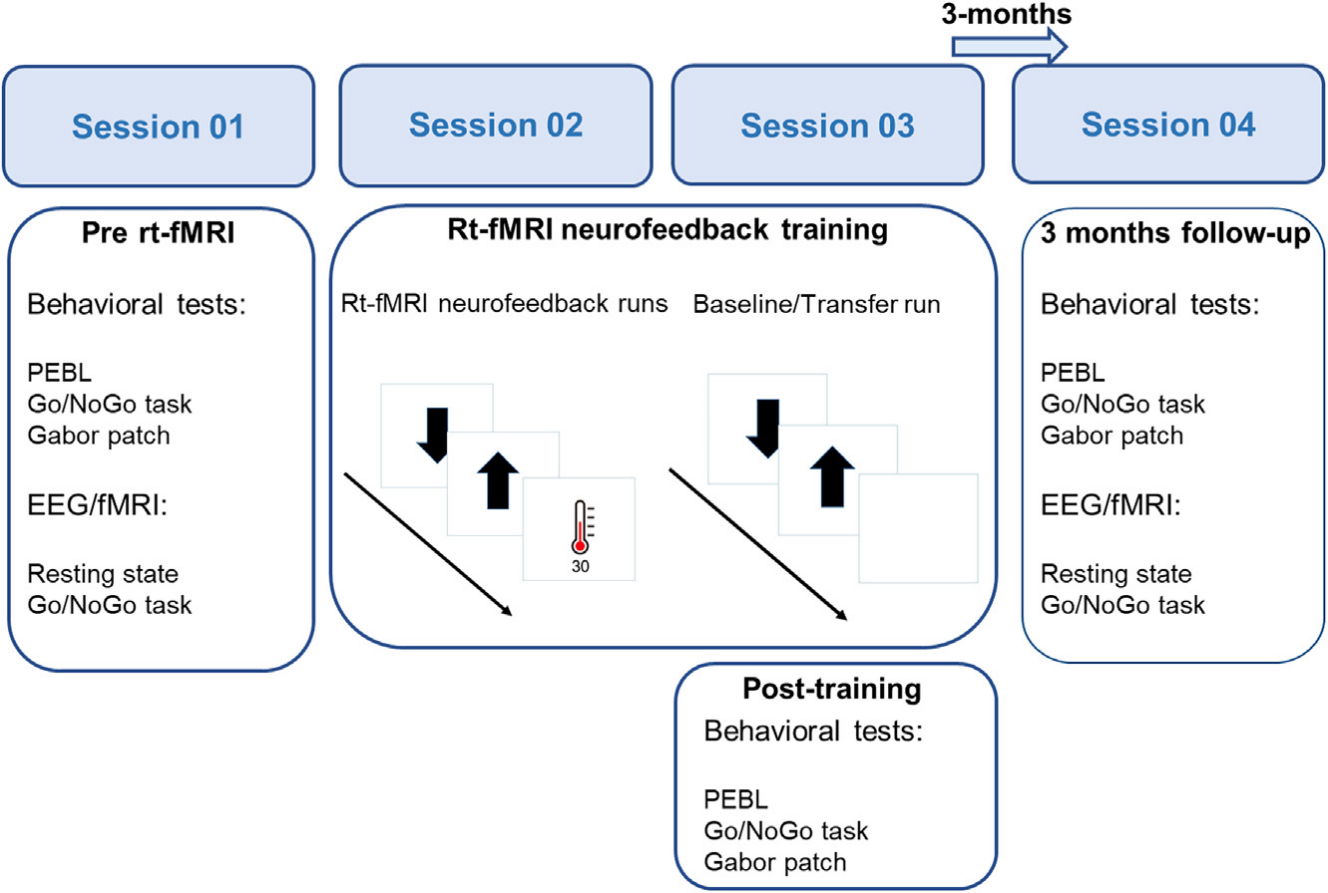
Study overview Participants in the rAIC and V1 group took part in four sessions. The first session included questionnaires, behavioral tests and a Go/NoGo task while EEG and fMRI were recorded simultaneously. Session two and three were rt-fMRI neurofeedback training sessions. For details about ROI placement see also Figure S1. After session three, participants again performed behavioral tests. Three months after neurofeedback training, participants repeated the procedure of the first study session. Participants in the mental-rehearsal group followed the same procedure but without the three months follow-up session and we did not record EEG-fMRI during session one.

We recruited 65 healthy participants who took part in two separate rt-fMRI neurofeedback training sessions embedded within four study sessions (see Figure 1 for a depiction of the experimental protocol). Participants were randomized to experimental or one of two control groups. The experimental group received feedback from their rAIC activity, while one control group received feedback from primary visual cortex area V1 (either from left V1 or from the right V1). A second control group performed the training sessions in the MRI scanner and was instructed to train mental strategies, which they think increase brain activity but without receiving any feedback. Attention and cognitive control measures were assessed before, just after and three months after the rt-fMRI neurofeedback training which allowed us to examine short- and long-term effects of rAIC neurofeedback training. By combining an implicit approach (not providing explicit strategies and information about the trained brain region to the participants) with two control groups we leveraged a neurofeedback design where we could control for known confound variables in rt-fMRI neurofeedback training^31^ and attribute changes in behavior to the rt-fMRI neurofeedback training. We hypothesized that only participants in the experimental group would learn to voluntarily increase rAIC activity with rt-fMRI neurofeedback training and consequently improve performance in cognitive control and attention tasks.

## Results

### Neural effects of rt-fMRI neurofeedback training

**Figure 2:**
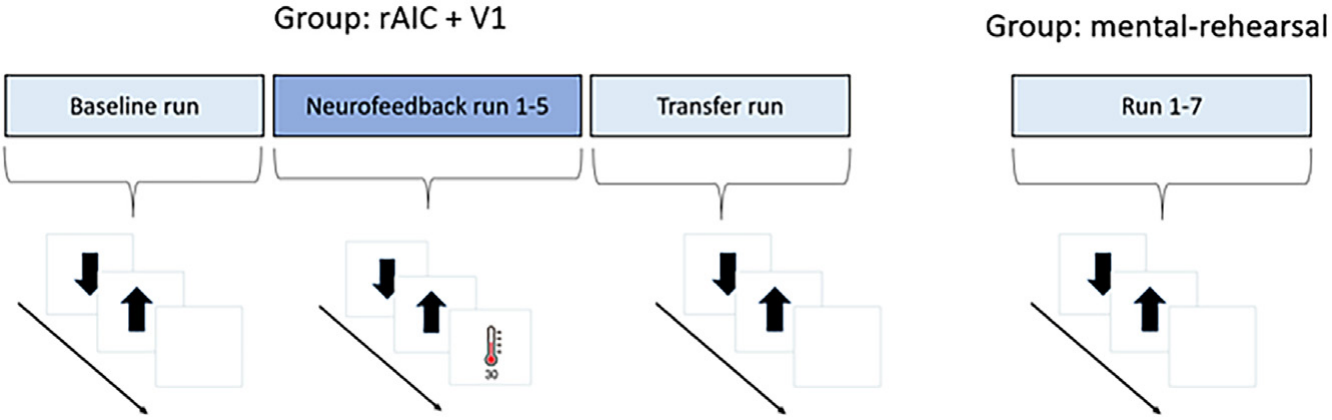
Schematic illustration of one session of rt-fMRI neurofeedback training for each group Participants in rAIC and V1 group performed at the beginning and at the end of each session runs without feedback (baseline/transfer). Between baseline and transfer run, they did five runs where they received neurofeedback training. Participants in the mental-rehearsal group conducted per session seven runs without feedback. For each participant, an anatomical MRI and a resting-state fMRI was recorded before the neurofeedback training.

**Figure 3:**
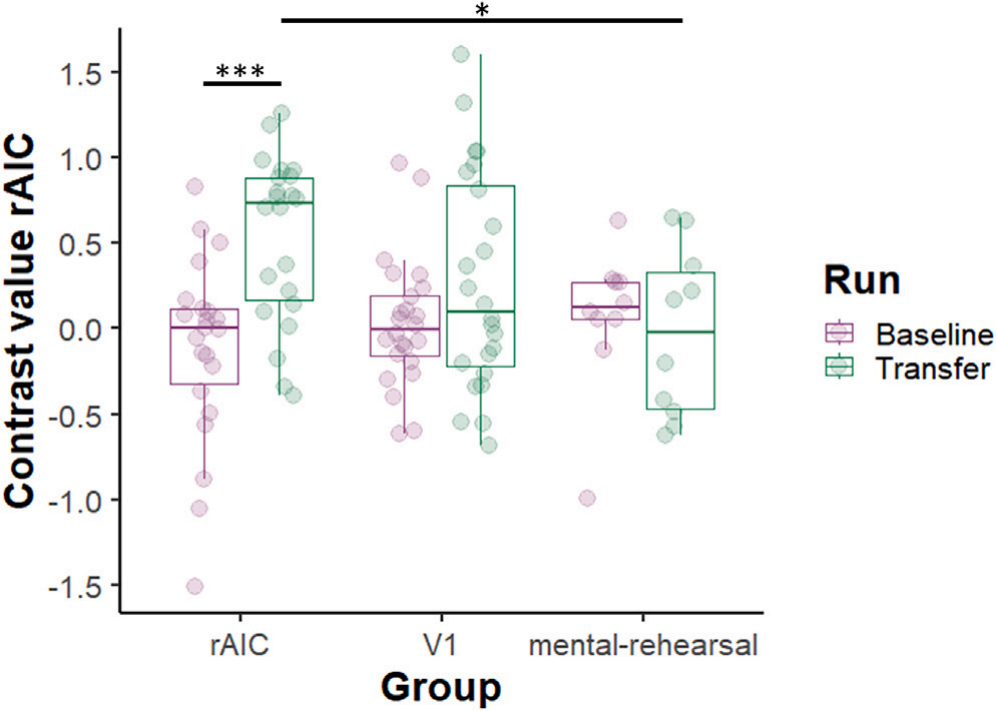
Activation level within rAIC before and after neurofeedback training Activity difference in contrast values (regulation > baseline) between the first baseline run and the last transfer run. Asterisks indicate significant paired t-test results: ∗p < 0.05, ∗∗p < 0.01, ∗∗∗p < 0.001. For rAIC activity over all runs see also Figures S2 and S3.

In line with a previous study, the overall regulation success was defined as the region of interest (ROI) activity difference between the last transfer run and the first baseline run (see Figure 2 for schematic illustration of the runs).^33^ A run (baseline, transfer) × group (rAIC, V1, mental-rehearsal) mixed ANOVA for the extracted rAIC contrast values (regulation > baseline) revealed a main effect of run (F_(1,53)_ = 5.68, p = 0.021) and a group × run interaction (F_(2,53)_ = 3.76, p = 0.03). The simple main effect of run was only significant for the rAIC group (F_(1,21)_ = 15, p = 0.002) and not for the V1 (F_(1,23)_ = 2.33, p = 0.42) or mental-rehearsal group (F_(1,9)_ = 0.17, p = 1). Pairwise paired t-test comparisons highlighted a significant increase from first baseline run to the last transfer run in the rAIC group (t(21) = 3.88, p = 0.0008, Cohen’s *d* = 1.28). No significant differences between runs were found in rAIC activity for the V1 (t(23) = 1.52, p = 0.14, Cohen’s *d* = 0.43), and mental-rehearsal groups (t(9) = 0.41, p = 0.68, Cohen’s *d* = −0.21). In addition, when splitting the V1 group into right and left V1 group, no significant differences between runs were found in rAIC activity for these groups (right V1 [rV1]: p = 0.07, Cohen’s *d* = −0.57, left V1 [lV1]: p = 0.81, Cohen’s *d =* −0.06). Baseline rAIC activity did not differ between groups (F_(2,53)_ = 0.85, p = 0.86). The activity during transfer runs did not differ between rAIC and V1 (p = 0.10). However, participants in the rAIC group showed increased activity during the transfer run compared to the mental-rehearsal group (t(17) = 3.03, p = 0.02, Cohen’s *d* = 1.16). RAIC activity during transfer run did not differ between V1 and mental-rehearsal group (p = 0.51) (see Figure 3).

To test whether participants in the V1 group were able to upregulate activity in their target ROI, we computed two-way ANOVA and linear regression for extracted V1 activity. For both, right and left V1 activity the two-way mixed ANOVA did not show any significant effect indicating no significant change from the first baseline run to the last transfer run independently of the feedback group.

Additionally, to examine ROI activity regulation success over the whole rt-fMRI neurofeedback training, linear regression of the extracted ROI activity over training runs was calculated. The rAIC group increased rAIC activity over the course of rt-fMRI neurofeedback runs (β = 0.04, R^2^ = 0.03, F_(1, 218)_ = 7.95, p = 0.005). While participants across V1 groups did not show a transfer effect for rAIC activity (F_(1,23)_ = 2.33, p = 0.42), participants in the right V1 group showed a trend for an increase in rAIC activity across rt-fMRI neurofeedback runs (β = 0.03, R^2^ = 0.03, F_(1,238)_ = 3.71, p = 0.056). When combining the two V1 groups a significant increase in rAIC activity over the course of rt-fMRI neurofeedback runs was observed (β = 0.03, R^2^ = 0.02, F_(1,238)_ = 6.16, p = 0.013). The mental-rehearsal group (β = −0.01, R^2^ = 0.003, F_(1, 98)_ = 0.37, p = 0.54) and the left V1 group (β = 0.03, R^2^ = 0.022, F_(1, 118)_ = 2.73, p = 0.1) did not show a linear learning effect (see Figures S2 and S3 in the supplemental information). For activity in right V1, the linear regression demonstrated a significant increase over the course of rt-fMRI neurofeedback runs for both the right V1 (β = 0.03, R^2^ = 0.04, F_(1, 118)_ = 4.37, p = 0.03) and the left V1 (β = 0.05, R^2^ = 0.05, F_(1, 118)_ = 6.94, p = 0.009) groups. Regarding activity extracted from left V1, only participants in the left V1 group increased activity over the course of neurofeedback runs (β = 0.05, R^2^ = 0.06, F_(1, 118)_ = 8.45, p = 0.004). Participants in the right V1 group did not significantly increase activity over runs in the left V1 (p = 0.09). In the rAIC and the mental-rehearsal group, the activity for both left (rAIC group p = 0.14, mental-rehearsal group p = 0.08) and right V1 (rAIC group p = 0.82, mental-rehearsal group p = 0.31) did not change. In addition, we analyzed activation in the whole V1 (right and left V1 mask merged). The two-way repeated measure ANOVA did not show any significant main effect or interaction effect. The linear regression revealed only for the right V1 (β = 0.03, R^2^ = 0.03, F_(1, 118)_ = 3.9, p = 0.05) and the left V1 (β = 0.05, R^2^ = 0.06, F_(1, 118)_ = 7.9, p = 0.005) group a significant increase of overall V1 activity across neurofeedback runs.

Furthermore, a whole-brain two-way (session × group) repeated measure ANOVA showed that compared to the V1 group, the rAIC group increased activity in the right supramarginal gyrus (54, −36, 32), right opercular part of the inferior parietal gyrus (50, 18, 8), rAIC (34, 8, 13), and left middle frontal gyrus (−35,34, 30) (voxel-level p < 0.001 uncorrected) during transfer run compared to baseline run (see Figure S4 in the supplemental information).

Participants reported trying a wide range of mental strategies to increase brain activity, common strategies included mental arithmetic (e.g., division, multiplication, or Fibonacci sequence) or emotional memories (e.g., thinking of sad, successful, or fearful situations). Importantly, strategies reported during transfer runs were similar to strategies used during the baseline runs. A list of strategies used during baseline and transfer runs is shown in supplemental information (Tables S1 and S2).

Together, these results demonstrate that participants in the rAIC group learned to upregulate activity in their rAIC over the course of neurofeedback runs and maintained self-regulation in the absence of feedback. Additionally, they increased activity in other brain regions associated with attention and cognitive control. None of these effects were observed in the other groups.

### Short and long-term attentional effects of rt-fMRI neurofeedback

We went on to test whether voluntary increase of rAIC activity resulted in improved performance in behavioral tests. Participants performed before (pre rt-fMRI), just after (post-training), and three months after the rt-fMRI neurofeedback training (3-month FU), a comprehensive test battery implemented in the Psychology Experiment Building Language (PEBL)^34^ containing the Attentional Network Task (ANT), a decision rule switching task (Switcher) and Corsi block task measuring visuo-spatial short-term working memory. Furthermore, a Go/NoGo and Gabor patch task were employed.

**Figure 4:**
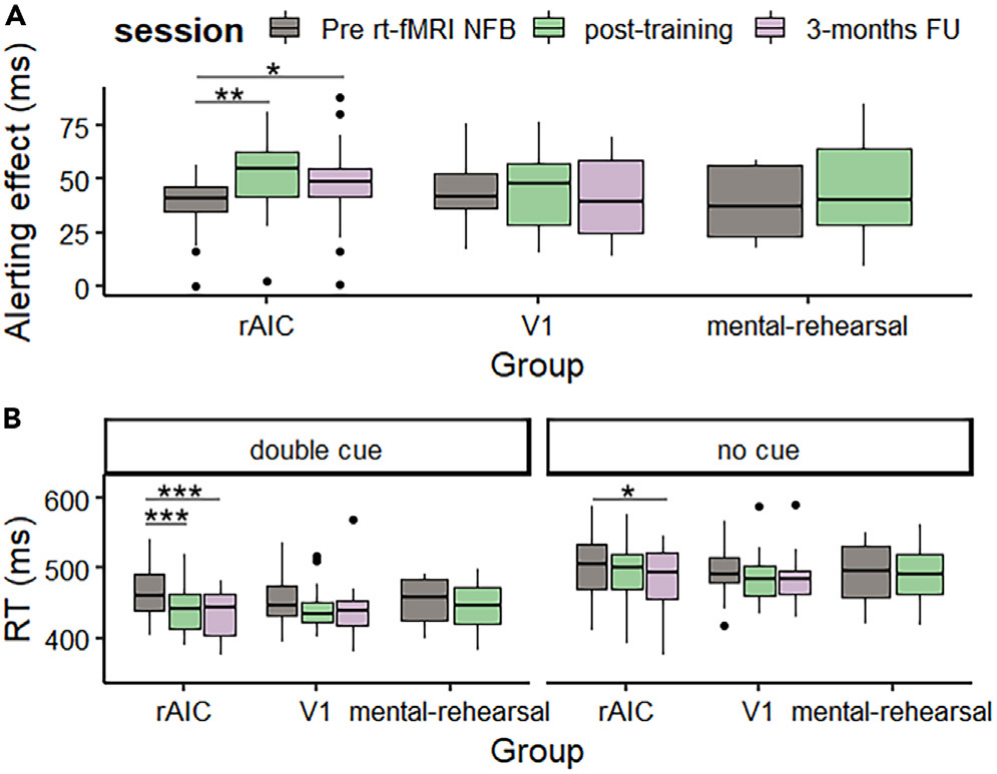
Alerting effects Participants in the rAIC group showed an increased alerting effect right after rt-fMRI neurofeedback training (post-training) compared to before (∗∗p < 0.01). This effect was also evident three months later (∗p < 0.05) (upper panel A). The lower panel B shows that this effect was driven by faster reaction times for the double cue condition for the examined time intervals. Post-hoc test showed that participants in the rAIC group responded significantly faster to double cue trials immediately after training compared to before rt-fMRI neurofeedback (∗∗∗p < 0.001). RTs remained shorter during the three months FU compared to before neurofeedback training (∗∗∗p < 0.001). RTs used for this figure were cleaned for outliers (see STAR Methods part). However, additional analysis was calculated without the values marked as outliers in the boxplots and can be found in the SI Figure S5 (results remained unchanged).

We used two-way mixed ANOVA with the factors group (rAIC, V1, mental-rehearsal) and session (pre rt-fMRI, post-training, 3-month FU) for each task to probe for effects of rt-fMRI neurofeedback training on behavior. Significant main effects and interactions were only found in the ANT task, which is a combination of a flanker and cueing task and simultaneously assesses alerting, orienting, and executive attention (see STAR Methods section for details). For the alerting component (reaction time (RT) no cue - double cue), a two-way mixed ANOVA with session as within-subject factor (pre rt-fMRI, post-training, 3-month FU) and group as between-subject factor (rAIC, V1, mental-rehearsal) revealed a main effect of session (F_(2,88)_ = 3.66, p = 0.03) and a group × session interaction (F_(2,88)_ = 3.92, p = 0.02). The post-hoc comparison showed a significant increase in alerting from pre rt-fMRI to post-training (t(21) = 4.1, p = 0.002, paired t-test Bonferroni corrected, Cohen’s d = 0.91) and 3-month FU (t(21) = 3.2, p = 0.01, paired t-test Bonferroni corrected, Cohen’s d = 0.58) for the rAIC group only (see Figure 4 upper plot A). To examine which condition drove the alerting effect we computed a three-way mixed ANOVA (group × session × cue type) which revealed a main effect of cue type. The no cue condition (506 ms ± 51.8 ms) exhibited a longer RT compared to the double cue condition (453 ms ± 47 ms). Furthermore, there was a main effect of sessions with pre rt-fMRI (491 ms ± 54 ms) > post-training (477 ms ± 57.6 ms) > 3-month FU (469 ms ± 54.8 ms) RT. Additionally to a significant session × cue type interaction (F_(2,88)_ = 3.6, p = 0.03) we found a significant three-way interaction between cue type, session, and group (F_(2,88)_ = 3.92, p = 0.02). The post-hoc comparison showed a significant decrease in double cue RT over sessions for the rAIC group only. RT decreased in the rAIC group from pre rt-fMRI to post-training (t(21) = −4.6, p = 0.0003, paired t-test Bonferroni corrected, Cohen’s d = 0.7) and from pre rt-fMRI to 3-month FU (t(21) = −4.8, p = 0.0002, paired t-test Bonferroni corrected, Cohen’s d = 0.8). Furthermore, the RT for no cue condition decreased from pre rt-fMRI to the 3-month FU session (t(21) = −2.9, p = 0.02, paired t-test Bonferroni corrected, Cohen’s d = 0.4) (see Figure 4 lower plot B).

For orienting attention, a three-way mixed ANOVA with cue type (spatial cue, central cue) and session as within-subject factors, plus group as a between-subject factor, was performed. The results revealed a main effect of session (F_(2, 88)_ = 13.13, p = 0.001) indicating faster RTs across sessions. Furthermore, the main effect of cue-type (F_(1, 44)_ = 265.607, p = 0.01) was significant which shows overall faster RT for spatial cues (428 ms ± 47.8 ms) compared to center cue (458 ms ± 49.3 ms).

For executive control, a three-way mixed ANOVA with flanker type (incongruent, congruent) and session as within-subject factors, plus group as a between-subject factor, revealed the main effect for session (F_(2, 88)_ = 17.28, p = 0.00003) and flanker type (F_(1, 44)_ = 703.747, p < 0.0001). Overall RTs decreased over sessions (pre rt-fMRI neurofeedback [495 ms ± 64.1 ms] > post-training [476 ms ± 60.8 ms] > 3-month FU [467 ms ± 57.5 ms]) and participants responded faster to congruent (443 ms ± 47.4 ms) compared to incongruent (517 ms ± 52.1 ms) trials. Moreover, a two-way interaction between session and flanker type (F_(2, 88)_ = 11.02, p = 0.00005) was found, indicating a smaller conflict effect for RTs across sessions.

Overall, these results show that participants increased alertness during an attention task, both short- and long-term, after rAIC rt-fMRI neurofeedback training. However, no rAIC specific neurofeedback training effect could be observed for the response inhibition, flanker, and spatial cuing task.

### Correlation analysis self-regulation and behavioral changes

We tested whether improvements in behavior were correlated with activity modulation in the rAIC following rt-fMRI neurofeedback training. Since only the alerting effect and especially the RT for double cue showed rt-fMRI group associated changes, exploratory correlations were performed with the regulation success for the rAIC activity (transfer effect, linear regression over rt-fMRI runs and last minus first rt-fMRI run). Correlating the transfer effect in rAIC activity with the change in RT for double cue from pre rt-fMRI neurofeedback training to the time point “post-training” did not show significant results in any of the examined groups. In addition, change in the overall alerting effect was not correlated to the extent of rAIC upregulation.

### Self-report and questionnaires

There was no significant difference between groups in the Hospital Anxiety and Depression Scale (HADS)^35^, Barratt Impulsiveness Scale (BIS-11)^36^ scores and motivation or attention during rt-fMRI neurofeedback sessions (Mann-Whitney U-tests p > 0.05). Furthermore, a one-way ANOVA revealed no significant group differences regarding age (F_(2,53)_ = 0.501, p = 0.61) or head motion (FD) (F_(2,53)_ = 0.055, p = 0.946). Nine out of ten participants in the mental-rehearsal group reported that they believe they performed real rt-fMRI neurofeedback training. The duration between rt-fMRI neurofeedback sessions was on average 2.75 days (SD 2.24 days). The duration for participants in the mental-rehearsal group (2.9 ± 2.51 days [M±SD], p = 0.86) and both V1 (lV1: 3 ± 2.61 days [M±SD], p = 0.98, rV1: 2.23 ± 1.96 days [M±SD], p = 0.46) groups was not different from the rAIC group (2.84 ± 2.17 days [M±SD]).

## Discussion

We used a longitudinal rt-fMRI design to target the rAIC with the goal of increasing cognitive control and attention based on neurofeedback. We found that participants were able to gain voluntary control over rAIC activity after two rt-fMRI neurofeedback training sessions in which they were provided with feedback about their ongoing rAIC activity. Furthermore, participants showed increased attention-related alertness during a speeded attention task, which persisted three months after the training. Our findings extend evidence for the critical role of the rAIC in attention-related alertness and provide initial insights into the potential of rt-fMRI neurofeedback training to enhance long-term specific components of attention in a variety of clinical conditions associated with rAIC dysfunction and attentional impairments.

The first goal of our study was to determine whether healthy participants can gain voluntary control over their rAIC activity without being instructed to use rAIC related strategies. To address this question, we split healthy participants in an experimental group receiving feedback about their rAIC activity and two control groups, one training to increase V1 activity and another not receiving any feedback during the neurofeedback sessions. Participants in the experimental group were able to modulate rAIC activity and maintain continuous increases of rAIC activity across runs spread over two separate days (within one week) during intermittent neurofeedback. Moreover, participants maintained their self-regulation after the second neurofeedback session even in the absence of neural feedback.

A meta-analysis by Emmert et al.^37^ identified the anterior insula as a key component of a regulatory network activated during neurofeedback training, regardless of the targeted area. Consistent with this, our study observed a tendency for heightened rAIC activity over runs in the V1 group. However, the absence of a transfer effect in both the V1 and mental-rehearsal groups suggests that the observed modulation of activity in the rAIC group was specifically tied to the feedback received during neurofeedback sessions, rather than the general process of regulation. This distinction highlights the unique impact of targeted feedback in the rAIC group.

Our implicit study design including two control groups allowed to exclude most of the known confounding factors in rt-fMRI neurofeedback studies.^31^^,^^38^ Specifically, with our mental-rehearsal group we tried to account for a possible effect of mental strategy training on cognitive control and attention, however, due to the fact that we did not provide a specific strategy to participants in the rAIC group (implicit rt-fMRI neurofeedback), and they applied a variety of mental strategies, it was not possible to provide control participants with a specific strategy. Nevertheless, control participants got the same instruction as the other participants and importantly, after the third session, nine out of ten participants believed that they did real rt-fMRI neurofeedback training even though they never saw a feedback signal. Furthermore, reported strategies during baseline and transfer runs were similar across the groups.

Participants in the V1 group were able to increase activity in their target ROI across rt-fMRI neurofeedback runs, yet they failed to transfer their self-regulation skills to the transfer run without feedback, which may explain the absence of changes in perceptual sensitivity for the Gabor patch task. This finding was unexpected since the feasibility of V1 regulation has been shown previously.^17^^,^^39^^,^^40^^,^^41^ However, in all these studies participants learned to regulate activity in a specific subpart of their V1 that was sensitive to visual stimulation during functional localizer runs. Since we did not use functional localizer in our study but rather presented participants with a feedback signal computed as average over a whole V1 in one hemisphere, the lack of self-regulation might be explained by low specificity of the feedback signal. Another reason could result from the small sample size due to the split in right and left V1 group (N = 12 for the right and left V1 groups, respectively). Our rt-fMRI neurofeedback findings are in line with previous studies showing modulation of rAIC activity as a consequence of neurofeedback training combined with explicit rAIC related emotion regulation-related mental strategies.^25^^,^^30^ Critically, we extended these findings by showing that participants can increase rAIC activity without knowing what brain region and behavior is intended to be changed.

The second goal of the present study was to assess the long-term impact of rAIC activity modulation on attention. We used the ANT task which is a combination of spatial cueing task^42^ and flanker task.^43^ The combination of cue and flanker conditions allow assessing three attentional subnetworks within a single task. Alerting attention is defined as the achieving and maintaining of intrinsic alertness (“readiness”) to respond to stimuli, while orienting refers to the selection/shifting of attention toward information among various sensory input and executive control is the process which allows resolving of conflict in mental operations and responses.^44^^,^^45^ Contrary to our initial hypothesis, our findings revealed significant effects solely on the alerting component of attention. Notably, improvements in alertness were sustained for at least three months following the modulation of rAIC activity, underscoring the robust nature of these specific cognitive enhancements (Figure 4). Our analysis revealed that the change in alertness was driven by faster RTs for double cue rather than longer RT for the no cue condition. This is a key finding, since larger alerting scores due to longer RT in the no cue condition would reflect difficulty in maintaining alertness.^46^ Thus, our findings suggest that participants in the rAIC group showed increased degrees of alertness after the neurofeedback training.

Notably, only the rAIC group showed a significant improvement in alertness right after neurofeedback training, which was maintained at three-month follow-up (Figure 4A). Likewise, even though RTs for double cue trials were faster in most groups (Figure 4B), this improved performance was strongest and only significant in the rAIC group. We did not observe a significant correlation between rAIC upregulation and individual improvements in alertness. This is consistent with reports that among the few clinical fMRI neurofeedback studies (28% of the studies analyzed) that report correlations, 35% found no significant correlation between regulatory success and behavioral changes.^47^ Future studies could benefit from incorporating network analysis across multiple brain regions, which may offer a more complete understanding of sources of individual differences underlying neurofeedback effects. In line with our behavioral findings, prior research has highlighted the important role of the rAIC and adjoining frontal operculum in the maintenance of alertness,^48^^,^^49^^,^^50^^,^^51^ and a recent lesion-symptom-mapping study in stroke patients reported that alerting effects, including RT for auditory warning cues, were significantly affected by lesions in the rAIC.^6^ Together, these results provide convergent evidence for the causal role of the rAIC in alertness and attention modulation.

Several behavioral intervention studies have attempted to modulate attention networks. However, meditation training^52^ and working memory training in ADHD^53^ did not reveal expected improvements in the alerting effect. Combining behavioral interventions with rAIC rt-fMRI neurofeedback may provide a more powerful approach for improving alertness and remediating diminished attentional abilities in a variety of disorders including schizophrenia,^54^ mild cognitive impairment,^55^ following chemotherapy,^56^ and ADHD.^57^ Thus, our result is of particular importance for advancing knowledge about the neurophysiological underpinning of attention and suggests an alternate way to improve alertness.

### Limitations of the study

One limitation of this study was the relative timing of the behavioral sessions. While the pre rt-fMRI behavioral tests were done at the beginning of the first session, the post-training behavioral tests were performed just after the second rt-fMRI neurofeedback session, meaning after having been ∼1.5 h in the MRI scanner. Since many participants reported that rt-fMRI neurofeedback training was tiring, our post-training behavioral effects might have been affected by participants' fatigue. Also, due to the single-blind design we cannot fully rule out a possible experimenter bias. Furthermore, although the sample size in our experimental rAIC and control V1 groups were higher than in other recent fMRI neurofeedback studies, findings from the smaller second mental-rehearsal control group should be interpreted with caution. Because this group did not show any effects right after training, we did not test them for long-term effects during follow-up. Finally, although the effects of alertness on the ANT task were specific to the rAIC neurofeedback group, we observed a general improvement for all groups in the two other components (orienting and executive control) of this task over sessions. In addition, rAIC neurofeedback did not lead to higher performance in Go/NoGo task performance compared to the V1 and mental-rehearsal groups. This observation was surprising, given the documented role of the rAIC in cognitive control processes associated with response inhibition. Further studies are required to address these limitations and clarify the behavioral implications of rAIC neurofeedback regulation.

### Conclusion

Our study provides evidence that healthy individuals can effectively self-regulate their rAIC activity using real-time fMRI neurofeedback training, even without explicit guidance, such as instructions on emotion regulation. Distinctly, participants receiving rAIC-targeted feedback exhibited significant and sustained increases in rAIC activity, surpassing the outcomes observed in two control groups. After neurofeedback training, participants showed long-lasting changes in attention-related alertness indicating a critical role of the rAIC in attentional processes. Our findings indicate that neurofeedback training focusing on rAIC upregulation can present a promising, non-invasive, and drug-free method for augmenting certain aspects of attentional capacities.

## STAR★Methods

### Key resources table

**Table:** 

REAGENT or RESOURCE	SOURCE	IDENTIFIER
**Deposited data**		

Behavioral and fMRI beta images	Zenodo	https://doi.org/10.5281/zenodo.10425813
Code for data analysis	Github on Zenodo	https://doi.org/10.5281/zenodo.8475

**Software and algorithms**		

MATLAB R2020a	MathWorks, Natick, MA	MathWorks – Entwickler von MATLAB und Simulink - MATLAB & Simulink
OpenNFT	OpenNFT · Open Neurofeedback Training	Article: https://doi.org/10.1016/j.neuroimage.2017.06.039
Sandwich Estimator toolbox	NISOx: SwE	Article: https://doi.org/10.1016/j.neuroimage.2014.03.029
Statistical Parametric Mapping (SPM12, v7771)	Wellcome Trust Centre for Neuroimaging	https://www.fil.ion.ucl.ac.uk/spm/
MRIcroGL	Chris Rorden	NITRC: MRIcroGL: Tool/Resource Info
R v4.1.2	R core team, 2022	R: The R Project for Statistical Computing (r-project.org)
WRS2 toolbox	Patrick Mair	CRAN - Package WRS2 (r-project.org)
PEBL: The Psychology Experimental Building Language	PEBL: The Psychology Experiment Building Language (sourceforge.net)	Article: https://doi.org/10.1016/j.jneumeth.2013.10.024
PsychoPy2	Open Science Tools Ltd	Article: https://doi.org/10.3758/s13428-018-01193-y

### Resource availability

#### Lead contact

Further information and requests for resources should be directed to and will be fulfilled by the lead contact, Jeanette Popovova (Jeanette.Popovova@gmail.com).

#### Materials availability

This study did not generate new unique reagents.

#### Data and code availability

• Relevant data has been deposited at Zenodo and is publicly available as of the date of publication. DOIs are listed in the key resources table.
• All original code has been deposited at Github and is publicly available as of the date of publication. DOIs are listed in the key resources table.
• Any additional information required to reanalyze the data reported in this paper is available from the lead contact upon request.

### Experimental model and study participant details

#### Participants

65 healthy right-handed young volunteers (23.83 ± 3.4 (mean (M) ± standard deviation (SD)) years old) with normal or corrected-to-normal visual acuity participated in this study. Participants were split into three different groups depending on the type of feedback they received during neurofeedback sessions. 28 participants were randomly assigned to the experimental rAIC feedback group, 27 participants to the V1 feedback control group, and 10 to the mental-rehearsal control group. Additionally, the V1 feedback group was split into participants receiving feedback from the right and left V1 with the aim of testing visual field specific changes in visual sensitivity following V1 upregulation. The first five participants had to be excluded from the analysis because of technical issues. Additionally, motion artefacts were too large for four participants (mean frame-wise displacement (FD) > 0.3mm) and their data was not included in the analysis. Thus, the final sample size was 56: 22 in the rAIC group (9 females, age 24.1 ± 3.4 (M ± SD)), 24 in the V1 (12 left V1 (6 females, age 23.7± 2.7 (M ± SD)), 12 right V1 (8 females, age 23.1± 3.7 (M ± SD))), and ten in the mental-rehearsal group (four females, age 24.3 ± 3.6 (M ± SD)). Exclusion criteria were left-handedness (assessed by Edinburgh Handedness Inventory – short form^58^), any psychiatric disorder, pregnancy, history of head injury, any metallic implants, and medication intake. Furthermore, participants had to speak and understand either German or English. Ancestry, race, or ethnicity were not assessed. All participants gave written informed consent before taking part in the study. This study is part of a large single-blind, placebo-controlled study which was approved by the local ethics committee of the Canton of Zurich in Switzerland (2017-00483) and registered at clinicalTrials.gov (https://clinicaltrials.gov, NCT04643340). Another part of this registered study was to examine the neuronal sources of both supraliminal and subliminal perception during a Go/NoGo task (on which we based our power analysis on) and to examine the impact of neurofeedback on supraliminal and subliminal perception and brain function (results will be reported elsewhere).

### Method details

#### Experimental overview

Recordings were performed from March 2020 until November 2021. The experimental protocol consisted of four sessions for the rAIC and V1 groups and three sessions for the mental-rehearsal control group (see Figure 1 for a depiction of the experimental protocol). The sessions were conducted on different days, the first three sessions within one week and the fourth session three months after the third session. All sessions for all participants were conducted at the MRI center of the psychiatric University Hospital in Zurich. During the first session, participants were asked to fill out the Hospital Anxiety and Depression (HADS)^35^ and the Baratt impulsiveness scale 11 (BIS-11)^36^ questionnaires. Furthermore, they performed a cognitive test battery containing tests on sustained attention, cognitive control, visuo-spatial short-term working memory, and visual perception tests (see paragraph attention test battery). After this, a resting-state sequence was recorded and participants performed four runs of the Go/NoGo task in the MRI scanner with simultaneous EEG-fMRI recording. The data presented here does not include any EEG-fMRI data and the protocol and analysis of this part of the study will therefore be presented elsewhere. During the second and third session of the experiment, rt-fMRI neurofeedback training was conducted. In order to evaluate the immediate effects of the neurofeedback training, participants were asked to perform the same test battery as during the first session, just after the rt-fMRI neurofeedback on the third session (post-training). Participants of the rAIC and V1 group took part in a three-month follow-up session where they underwent the same cognitive test battery and EEG-fMRI recordings.

#### MRI imaging parameters

The MRI images were acquired on a 3 Tesla MRI scanner (Philips Achieva, upgraded to dStream platform), equipped with a 32-channel receive head coil. Functional images for rt-fMRI neurofeedback were acquired with a T2∗-weighted gradient-echo-planar sequence with a repetition time (TR) = 2000 ms, echo time (TE) = 35 ms, flip angle = 82°, FOV = 220 mm × 220 mm, voxel size = 2 × 2 × 4 mm^3^, matrix size = 112 x 112, and 27 slices per volume with whole-brain coverage. 170 functional images were collected during each neurofeedback run (duration = 5.8 min). Anatomical images were collected with a 3D MPRAGE sequence: TR = 9.32 ms, TEs = 4.59 ms, flip angle = 8°, FOV = 240 mm × 240 mm, voxel size = 1 × 1 × 1 mm3, 160 slices, and duration = 3.7 min.

#### Neurofeedback protocol

Participants in the rAIC and V1 groups underwent two sessions of rt-fMRI neurofeedback training on two different days within a week and during similar daytime (8 am to 1 pm). On each day, participants performed five neurofeedback runs leading to a total number of ten training runs across all sessions. Additionally, in order to assess participants’ ability to regulate region-of-interest (ROI) activity in the absence of feedback, so-called baseline and transfer runs were conducted at the beginning and at the end of each session, respectively. Before neurofeedback runs, anatomical images and resting-state fMRI were acquired. During the resting-state sequence, participants were asked to fixate on a central white cross, presented on a grey screen. Neurofeedback runs consisted of five blocks composed of a 20 s (10 TRs) baseline condition, 40 s (20 TRs) regulation condition, and 4 s (2 TRs) feedback presentation (see Figure 2). During the baseline condition, a black downward arrow was displayed, and participants were asked to count backward from 100 in steps of two. This is a common procedure in rt-fMRI neurofeedback studies to assure that participants maintain stable baseline activity.^18^^,^^59^

During the regulation condition, a black upwards arrow was presented. Here, participants were instructed to increase their brain activity using any mental strategy they thought might work. Participants were told that the scale of the subsequently presented feedback reflected how well they upregulated brain activity and that they should try to make the scale rise as high as possible. Participants were free to choose any mental strategy they want. However, we stressed that they should not use simple counting since the feedback was calculated as the difference in brain activation between regulation and baseline block. In addition, participants were asked to not change their strategy within a run, however, they were free to change or adapt their strategy between runs. After each run, participants were asked to report the strategy they used. Furthermore, we asked them to rate the success of the used strategy to control the feedback on a scale from 1 (very bad) to 5 (very good). Visual feedback was presented in form of a thermometer icon with the temperature scale representing the difference in ROI activity between the previous regulation and baseline block (= average feedback value). The thermometer scale had ten positive and one zero levels and was color-coded in blue (level 0 to 2), violet (3 to 6), and red (7 to 10). In addition, below the thermometer icon, an integer ranging from 0 to 50 (thermometer level multiplied by 5) was presented to indicate the temperature reading numerically. In order to help participants to decide whether they should keep or change the strategy between runs, at the end of each neurofeedback run we presented them with their average feedback value (transposed to average points, see above) for the corresponding run.

During baseline and transfer runs, a white blank was presented instead of a thermometer icon. This difference aside, these runs were identical to the neurofeedback runs. These runs were included to assess whether participants showed a transfer effect meaning if they were able to maintain the ability to regulate ROI activation in the absence of feedback.^19^^,^^60^ During transfer runs, participants were asked to use the strategy that overall worked best for them. The mental-rehearsal control group performed 14 runs identical to the baseline/transfer run. Participants in this group were told that they participated in a rt-fMRI neurofeedback study, and they received the same instructions regarding the baseline and regulation condition, however, we did not inform them about the existence of the other two groups or a feedback condition. For each neurofeedback session, we assessed how motivated and attentive participants were during the training (self-report Likert scale from one to five). Additionally, after the second neurofeedback session, we asked the participants in the mental-rehearsal control group if they believed that they participated in a real rt-fMRI neurofeedback training. Each neurofeedback training lasts approximately 60 minutes. The whole session with instructions, pre-recordings and rt-fMRI neurofeedback training lasts around 90 minutes.

#### Real-time fMRI setup and feedback calculation

At the beginning of each session, MNI (Montreal Neurological Institute)-based ROI templates (taken from the Willard functional ROIs atlas (http://findlab.stanford.edu/functional_ROIs.html), for details see Figure S1) were transformed into the participant’s native space (T1-weighted structural scan) using Statistical Parametric Mapping 12 (SPM12; Wellcome Trust Centre for Neuroimaging London, United Kingdom) and custom-made MATLAB scripts. This procedure ensured that the same ROIs were targeted across the two different training days. During neurofeedback and baseline/transfer runs, acquired and reconstructed functional data was transferred from the MRI PC to a separate stimulation PC where the data was preprocessed and analyzed online using OpenNFT.^61^ Preprocessing included realignment, reslicing, and spatial smoothing with an isotropic Gaussian kernel with a 5-mm full width at half maximum (FWHM). Furthermore, the time course from the ROI was extracted and signal drift, spikes, and high-frequency noise were removed. Finally, the feedback signal was calculated as the difference in percent signal change between regulation and baseline condition for each block separately. The neurofeedback stimuli and feedback were presented to the participants through MR-compatible video goggles (Resonance Technology Inc., USA) using a custom-made script in PsychoPy2.^62^

#### Attention test battery

In order to evaluate whether there were effects of the rt-fMRI neurofeedback training on attention, participants performed the ANT (implemented the PEBL^34^) before and after the rt-fMRI neurofeedback training. The ANT is a cognitive flanker task with different cues and flanker conditions which allows assessing three components of attention: *orienting, alerting and executive control*. Each trial starts with a fixation cross which is presented for the whole trial. Participants are instructed to report the direction (left or right) of the target. The target is a central arrow pointing leftward or rightward and can be surrounded by two flankers on each side and presented either above or below the fixation cross. The flankers are either congruent (same direction as the target) or incongruent (point in the opposite direction of the target), in the neutral condition there are no flankers. Before the target occurs, there is either no cue or one of three types of warning cues. In the centre cue condition, the cue is presented on the fixation cross, in the double cue condition the warning cues are above and below the central fixation and in the spatial cue condition the cue is either above or below the cross indicating where the target will be presented (100% valid). The no cue and centre cue condition are control conditions, the double cue condition measures alerting, and the spatial cue orienting. Executive control is measured by the incongruent vs. congruent flanker condition. The ANT served as our primary outcome measure because it requires several mental processes ranging from the implementation of attention and cognitive control to the resolution of conflict, which may be all dependent on rAIC activity.

#### Other cognitive measures

We also examined secondary cognitive measures which assess different aspects of attention and cognitive control such as task-switching (Switcher) and location memory-span (Corsi block-tapping test).^63^ Furthermore, inspired by the finding that the rAIC seems to be involved in early unconscious cognitive control implementation^16^ we also administered a Go/NoGo task with supraliminal and subliminal Go and NoGo cues. We used an adapted version of the Go/NoGo task used in Van Gaal et al.^16^ Lastly, to test for possible effects of V1 rt-fMRI neurofeedback training on visual sensitivity we used an orientation discrimination task. During the task, participants had to indicate the orientation of Gabor patches (right or left), which illustrated a circular sinewave raster that had a gradual Gaussian blur edge.

### Quantification and statistical analysis

#### FMRI analysis

All functional MRI images were analyzed using MATLAB R2020a and SPM12. Preprocessing for each rt-fMRI neurofeedback run, included slice-time correction, realignment to the first scan of the session, co-registration of the functional to the anatomical image, segmentation, normalization into MNI space and spatial smoothing with a Gaussian kernel of 8-mm FWHM.

For the first level GLM analysis, the baseline, regulation, and feedback periods were modelled as boxcar functions and convolved with the canonical hemodynamic function of SPM. To get equal condition length and because OpenNFT is only considering the last six TRs for feedback calculation,^61^ we considered the last 10 TRs for the regulation condition and the entire 10 TRs for the baseline condition. Furthermore, six motion parameters were included as regressors of no interest. For each run the contrast ‘regulation > baseline’ was computed.

For the ROI analysis, we extracted for each participant from each ROI (rAIC, right and left V1) the average values from contrast images (regulation > baseline) of the first-level analysis of each run, using custom made MATLAB scripts. The overall regulation success was defined before data collection as the difference between the last transfer run and the first baseline run.^33^ We will refer to this effect as the transfer effect. In order to evaluate whether participants would show such a transfer effect for their trained ROI, a two-way mixed analysis of variance (ANOVA) with run (first baseline run versus last transfer run) as within factor and group (rAIC, right and left V1, and mental-rehearsal) as between factor was computed for “activation” in each ROI. For significant ANOVA effects, we computed post-hoc tests using pairwise comparisons. Bonferroni correction was used to correct for multiple testing. Cohen’s d was computed as measure of effect size for the posthoc pairwise comparisons results. Additionally, to test whether participants showed a linear activation increase with increasing number of rt-fMRI neurofeedback runs in the trained ROI but not in the other ROI, contrast values for the ten rt-fMRI neurofeedback runs were fitted to a linear regression for each group and ROI independently.

In addition to the ROI-specific analyses, we investigated the change in activation across the whole brain using second-level whole-brain random-effects analysis. For this, the contrast image (regulation > baseline) for the first baseline run and the last transfer run were submitted to second-level analysis for each group separately using paired t-test. Contrast maps were thresholded using a voxel-threshold of *p* < 0.001 and corrected for multiple comparison using false discovery rate (FDR) on cluster level *p* < 0.05. In addition, to test for group × session interaction for whole brain activation, a 2 × 2 factorial design including the group (rAIC versus V1) and session (first baseline run versus last transfer run) was modelled using the Sandwich Estimator (SwE) method as implemented in the SwE toolbox (http://warwick.ac.uk/tnichols/SwE).^64^ The SwE toolbox is used to analyse repeated measure fMRI data by fitting a marginal model by using a non-iterative (sandwich estimator) method to estimate the population model. For this analysis, only the subjects from the rAIC and V1 (all participants receiving V1 neurofeedback) groups were included to guarantee equal sample size between groups. We used MRIcroGL (NITRC: MRIcroGL: Tool/Resource Info) to visualize second-level brain activation maps.

#### Attention and cognitive measures

*ANT* Accuracy and RT were extracted and computed for each attentional network (orienting, alerting, and executive control). Trial labelled as outliers in RT (values that lie outside the 2.5 and 97.5 percentiles interval) were identified and eliminated (< 7% of the data). As proposed by Fan et al.,^44^ the alerting effect is calculated by subtracting the mean RT of double cue condition from the mean RT of the no cue condition. For the orienting effect, the mean RT of the spatial cue conditions was subtracted from the mean RT of the centre cue. The conflict effect (executive control) was computed as the mean RT of all congruent flanking conditions subtracted from the mean of incongruent flanking condition. For each component (alerting, orienting, executive control), we computed a two-way mixed ANOVA with session (pre rt-fMRI, post-training and 3-months follow-up) as within-participants variable and group (rAIC, V1 and mental-rehearsal) as between-participants variable. Following significant ANOVA results, differences between groups and sessions were analyzed post-hoc using pairwise comparison. Bonferroni correction was used to correct for multiple testing. Additionally, Pearson correlation was computed between the change in attentional network (effect for each subnetwork) and the change in rAIC activation due to rt-fMRI neurofeedback training (i.e., the difference in rAIC activation (regulation-baseline) from first baseline run to last transfer run).

Before conducting statistical tests (for behavioral data and fMRI ROI analysis), assumptions of the ANOVA test were checked and if needed non-parametric tests were used. All statistical analyses were done using R version 4.1.2. For non-parametric ANOVA we used the robust mixed ANOVA implemented in the WRS2 package in R. Scripts used for all data analysis are made available on the Github repository: https://github.com/popovov7/rtfMRI_rAIC. To assure valid reporting of rt-fMRI neurofeedback results we followed the CRED-nf checklist.^65^ The checklist (Table S3) can be found in the supplemental information.

### Additional resources

This study is part of a large single-blind, placebo-controlled study which was approved by the local ethics committee of the Canton of Zurich in Switzerland (2017-00483) and registered at clinicalTrials.gov (https://clinicaltrials.gov, NCT04643340).

## References

[1] King J.A., Colla M., Brass M., Heuser I., von Cramon D. (2007). Inefficient cognitive control in adult ADHD: evidence from trial-by-trial Stroop test and cued task switching performance. Behav. Brain Funct..

[2] Edwards B.G., Barch D.M., Braver T.S. (2010). Improving prefrontal cortex function in schizophrenia through focused training of cognitive control. Front. Hum. Neurosci..

[3] Larson M.J., Perlstein W.M., Demery J.A., Stigge-Kaufman D.A. (2006). Cognitive control impairments in traumatic brain injury. J. Clin. Exp. Neuropsychol..

[4] Cavanagh J.F., Ryman S., Richardson S.P. (2022). Cognitive control in Parkinson’s disease. Prog. Brain Res..

[5] Seeley W.W., Menon V., Schatzberg A.F., Keller J., Glover G.H., Kenna H., Reiss A.L., Greicius M.D. (2007). Dissociable intrinsic connectivity networks for salience processing and executive control. J. Neurosci..

[6] Cazzoli D., Kaufmann B.C., Paladini R.E., Müri R.M., Nef T., Nyffeler T. (2021). Anterior insula and inferior frontal gyrus: where ventral and dorsal visual attention systems meet. Brain Commun..

[7] Menon V., Uddin L.Q. (2010). Saliency, switching, attention and control: a network model of insula function. Brain Struct. Funct..

[8] Molnar-Szakacs I., Uddin L.Q. (2022). Anterior insula as a gatekeeper of executive control. Neurosci. Biobehav. Rev..

[9] Nelson S.M., Dosenbach N.U.F., Cohen A.L., Wheeler M.E., Schlaggar B.L., Petersen S.E. (2010). Role of the anterior insula in task-level control and focal attention. Brain Struct. Funct..

[10] Sridharan D., Levitin D.J., Menon V. (2008). A critical role for the right fronto-insular cortex in switching between central-executive and default-mode networks. Proc. Natl. Acad. Sci. USA.

[11] Cai W., Ryali S., Chen T., Li C.-S.R., Menon V. (2014). Dissociable Roles of Right Inferior Frontal Cortex and Anterior Insula in Inhibitory Control: Evidence from Intrinsic and Task-Related Functional Parcellation, Connectivity, and Response Profile Analyses across Multiple Datasets. J. Neurosci..

[12] Cai W., Chen T., Ryali S., Kochalka J., Li C.-S.R., Menon V. (2016). Causal Interactions Within a Frontal-Cingulate-Parietal Network During Cognitive Control: Convergent Evidence from a Multisite–Multitask Investigation. Cerebr. Cortex.

[13] Dosenbach N.U.F., Visscher K.M., Palmer E.D., Miezin F.M., Wenger K.K., Kang H.C., Burgund E.D., Grimes A.L., Schlaggar B.L., Petersen S.E. (2006). A Core System for the Implementation of Task Sets. Neuron.

[14] Dosenbach N.U.F., Fair D.A., Miezin F.M., Cohen A.L., Wenger K.K., Dosenbach R.A.T., Fox M.D., Snyder A.Z., Vincent J.L., Raichle M.E. (2007). Distinct brain networks for adaptive and stable task control in humans. Proc. Natl. Acad. Sci. USA.

[15] Spielberg J.M., Miller G.A., Heller W., Banich M.T. (2015). Flexible brain network reconfiguration supporting inhibitory control. Proc. Natl. Acad. Sci. USA.

[16] Van Gaal S., Ridderinkhof K.R., Scholte H.S., Lamme V.A.F. (2010). Unconscious activation of the prefrontal no-go network. J. Neurosci..

[17] Scharnowski F. (2012).

[18] Young K.D., Zotev V., Phillips R., Misaki M., Yuan H., Drevets W.C., Bodurka J. (2014). Real-Time fMRI Neurofeedback Training of Amygdala Activity in Patients with Major Depressive Disorder. PLoS One.

[19] Koush Y., Meskaldji D.-E., Pichon S., Rey G., Rieger S.W., Linden D.E.J., Van De Ville D., Vuilleumier P., Scharnowski F. (2017). Learning Control Over Emotion Networks Through Connectivity-Based Neurofeedback. Cerebr. Cortex.

[20] Pamplona G.S.P., Heldner J., Langner R., Koush Y., Michels L., Ionta S., Scharnowski F., Salmon C.E.G. (2020). Network-based fMRI-neurofeedback training of sustained attention. Neuroimage.

[21] Ramot M., Kimmich S., Gonzalez-Castillo J., Roopchansingh V., Popal H., White E., Gotts S.J., Martin A. (2017). Direct modulation of aberrant brain network connectivity through real-time NeuroFeedback. Elife.

[22] Zilverstand A., Sorger B., Slaats-Willemse D., Kan C.C., Goebel R., Buitelaar J.K. (2017). fMRI Neurofeedback Training for Increasing Anterior Cingulate Cortex Activation in Adult Attention Deficit Hyperactivity Disorder. An Exploratory Randomized, Single-Blinded Study. PLoS One.

[23] Alegria A.A., Wulff M., Brinson H., Barker G.J., Norman L.J., Brandeis D., Stahl D., David A.S., Taylor E., Giampietro V., Rubia K. (2017). Real-time fMRI neurofeedback in adolescents with attention deficit hyperactivity disorder. Hum. Brain Mapp..

[24] deBettencourt M.T., Cohen J.D., Lee R.F., Norman K.A., Turk-Browne N.B. (2015). Closed-loop training of attention with real-time brain imaging. Nat. Neurosci..

[25] Caria A., Veit R., Sitaram R., Lotze M., Weiskopf N., Grodd W., Birbaumer N. (2007). Regulation of anterior insular cortex activity using real-time fMRI. Neuroimage.

[26] Kanel D., Al-Wasity S., Stefanov K., Pollick F.E. (2019). Empathy to emotional voices and the use of real-time fMRI to enhance activation of the anterior insula. Neuroimage.

[27] Lawrence E.J., Su L., Barker G.J., Medford N., Dalton J., Williams S.C.R., Birbaumer N., Veit R., Ranganatha S., Bodurka J. (2014). Self-regulation of the anterior insula: Reinforcement learning using real-time fMRI neurofeedback. Neuroimage.

[28] Yao S., Becker B., Geng Y., Zhao Z., Xu X., Zhao W., Ren P., Kendrick K.M. (2016). Voluntary control of anterior insula and its functional connections is feedback-independent and increases pain empathy. Neuroimage.

[29] Buyukturkoglu K., Roettgers H., Sommer J., Rana M., Dietzsch L., Arikan E.B., Veit R., Malekshahi R., Kircher T., Birbaumer N. (2015). Self-Regulation of Anterior Insula with Real-Time fMRI and Its Behavioral Effects in Obsessive-Compulsive Disorder: A Feasibility Study. PLoS One.

[30] Ruiz S., Lee S., Soekadar S.R., Caria A., Veit R., Kircher T., Birbaumer N., Sitaram R. (2013). Acquired self-control of insula cortex modulates emotion recognition and brain network connectivity in schizophrenia. Hum. Brain Mapp..

[31] Sorger B., Scharnowski F., Linden D.E.J., Hampson M., Young K.D. (2019). Control freaks: Towards optimal selection of control conditions for fMRI neurofeedback studies. Neuroimage.

[32] Watanabe T., Sasaki Y., Shibata K., Kawato M. (2017). Advances in fMRI Real-Time Neurofeedback. Trends Cognit. Sci..

[33] Young K.D., Siegle G.J., Zotev V., Phillips R., Misaki M., Yuan H., Drevets W.C., Bodurka J. (2017). Randomized Clinical Trial of Real-Time fMRI Amygdala Neurofeedback for Major Depressive Disorder: Effects on Symptoms and Autobiographical Memory Recall. Am. J. Psychiatr..

[34] Mueller S.T., Piper B.J. (2014). The Psychology Experiment Building Language (PEBL) and PEBL Test Battery. J. Neurosci. Methods.

[35] Stern A.F. (2014). The Hospital Anxiety and Depression Scale. Occup. Med..

[36] Reise S.P., Moore T.M., Sabb F.W., Brown A.K., London E.D. (2013). The Barratt Impulsiveness Scale - 11: Reassessment of its Structure in a Community Sample. Psychol. Assess..

[37] Emmert K., Kopel R., Sulzer J., Brühl A.B., Berman B.D., Linden D.E.J., Horovitz S.G., Breimhorst M., Caria A., Frank S. (2016). Meta-analysis of real-time fMRI neurofeedback studies using individual participant data: How is brain regulation mediated?. Neuroimage.

[38] Lubianiker N., Goldway N., Fruchtman-Steinbok T., Paret C., Keynan J.N., Singer N., Cohen A., Kadosh K.C., Linden D.E.J., Hendler T. (2019). Process-based framework for precise neuromodulation. Nat. Human Behav..

[39] Andersson P., Ragni F., Lingnau A. (2019). Visual imagery during real-time fMRI neurofeedback from occipital and superior parietal cortex. Neuroimage.

[40] Scharnowski F., Rosa M.J., Golestani N., Hutton C., Josephs O., Weiskopf N., Rees G. (2014). Connectivity Changes Underlying Neurofeedback Training of Visual Cortex Activity. PLoS One.

[41] Shibata K., Watanabe T., Sasaki Y., Kawato M. (2011). Perceptual learning incepted by decoded fMRI neurofeedback without stimulus presentation. Science.

[42] Posner M.I. (1980). Orienting of Attention. Q. J. Exp. Psychol..

[43] Eriksen B.A., Eriksen C.W. (1974). Effects of noise letters upon the identification of a target letter in a nonsearch task. Percept. Psychophys..

[44] Fan J., McCandliss B.D., Sommer T., Raz A., Posner M.I. (2002). Testing the Efficiency and Independence of Attentional Networks. J. Cognit. Neurosci..

[45] Posner M.I., Petersen S.E. (1990). The attention system of the human brain. Annu. Rev. Neurosci..

[46] Posner M.I. (2008). Measuring Alertness. Ann. N. Y. Acad. Sci..

[47] Tursic A., Eck J., Lührs M., Linden D.E.J., Goebel R. (2020). A systematic review of fMRI neurofeedback reporting and effects in clinical populations. Neuroimage Clin..

[48] Clemens B., Zvyagintsev M., Sack A.T., Heinecke A., Willmes K., Sturm W. (2011). Revealing the Functional Neuroanatomy of Intrinsic Alertness Using fMRI: Methodological Peculiarities. PLoS One.

[49] Coste C.P., Kleinschmidt A. (2016). Cingulo-opercular network activity maintains alertness. Neuroimage.

[50] Daly S., Thai J., Belkhiria C., Langley C., Le Blanche A., de Marco G. (2020). Temporal Deployment of Attention by Mental Training: an fMRI Study. Cognit. Affect Behav. Neurosci..

[51] Sadaghiani S., D’Esposito M. (2015). Functional Characterization of the Cingulo-Opercular Network in the Maintenance of Tonic Alertness. Cerebr. Cortex.

[52] Tang Y.-Y., Ma Y., Wang J., Fan Y., Feng S., Lu Q., Yu Q., Sui D., Rothbart M.K., Fan M., Posner M.I. (2007). Short-term meditation training improves attention and self-regulation. Proc. Natl. Acad. Sci. USA.

[53] Dotare M., Bader M., Mesrobian S.K., Asai Y., Villa A.E.P., Lintas A. (2020). Attention Networks in ADHD Adults after Working Memory Training with a Dual n-Back Task. Brain Sci..

[54] Amado I., Lupiañez J., Chirio M., Landgraf S., Willard D., Olié J.P.J.P., Krebs M.O. (2011). Alertness can be improved by an interaction between orienting attention and alerting attention in schizophrenia. Behav. Brain Funct..

[55] Jeong E., Cha K.S., Shin H.-R., Kim E.Y., Jun J.-S., Kim T.-J., Byun J.-I., Shin J.-W., Sunwoo J.-S., Jung K.-Y. (2022). Alerting network alteration in isolated rapid eye movement sleep behavior disorder patients with mild cognitive impairment. Sleep Med..

[56] Chen X., He X., Tao L., Cheng H., Li J., Zhang J., Qiu B., Yu Y., Wang K. (2017). The attention network changes in breast cancer patients receiving neoadjuvant chemotherapy: Evidence from an arterial spin labeling perfusion study. Sci. Rep..

[57] Lecendreux M., Konofal E., Bouvard M., Falissard B., Mouren-Siméoni M.C. (2000). Sleep and alertness in children with ADHD. JCPP (J. Child Psychol. Psychiatry).

[58] Veale J.F. (2014). Edinburgh Handedness Inventory - Short Form: a revised version based on confirmatory factor analysis. Laterality.

[59] Robineau F., Rieger S.W., Mermoud C., Pichon S., Koush Y., Van De Ville D., Vuilleumier P., Scharnowski F. (2014). Self-regulation of inter-hemispheric visual cortex balance through real-time fMRI neurofeedback training. Neuroimage.

[60] Zotev V., Krueger F., Phillips R., Alvarez R.P., Simmons W.K., Bellgowan P., Drevets W.C., Bodurka J. (2011). Self-Regulation of Amygdala Activation Using Real-Time fMRI Neurofeedback. PLoS One.

[61] Koush Y., Ashburner J., Prilepin E., Sladky R., Zeidman P., Bibikov S., Scharnowski F., Nikonorov A., De Ville D.V. (2017). OpenNFT: An open-source Python/Matlab framework for real-time fMRI neurofeedback training based on activity, connectivity and multivariate pattern analysis. Neuroimage.

[62] Peirce J., Gray J.R., Simpson S., MacAskill M., Höchenberger R., Sogo H., Kastman E., Lindeløv J.K. (2019). PsychoPy2: Experiments in behavior made easy. Behav. Res. Methods.

[63] Kessels R.P., van Zandvoort M.J., Postma A., Kappelle L.J., de Haan E.H. (2000). The Corsi Block-Tapping Task: standardization and normative data. Appl. Neuropsychol..

[64] Guillaume B., Hua X., Thompson P.M., Waldorp L., Nichols T.E., Alzheimer's Disease Neuroimaging Initiative (2014). Fast and accurate modelling of longitudinal and repeated measures neuroimaging data. Neuroimage.

[65] Ros T., Enriquez-Geppert S., Zotev V., Young K.D., Wood G., Whitfield-Gabrieli S., Wan F., Vuilleumier P., Vialatte F., Van De Ville D. (2020). Consensus on the reporting and experimental design of clinical and cognitive-behavioural neurofeedback studies (CRED-nf checklist). Brain.

