# Supplementary material for: Enhanced attention-related alertness following right anterior insular cortex neurofeedback training
Source: iScience. 2024 Jan 15;27(2):108915. doi: 10.1016/j.isci.2024.108915 (PMC10839684; doi:10.1016/j.isci.2024.108915)
Supplement: Document S1. Figures S1–S5 and Tables S1–S3 [file mmc1.pdf]

iScience, Volume *27*

Supplemental information

Enhanced attention-related alertness following right anterior insular cortex neurofeedback training

Jeanette Popovova, Reza Mazloum, Gianluca Macauda, Philipp Stämpfli, Patrik Vuilleumier, Sascha Frühholz, Frank Scharnowski, Vinod Menon, and Lars Michels

Supplementary Information

1. Supplementary Figures

Figure S1. ROI definition for rt-fMRI neurofeedback (related to Figure 1 and STAR Method section “Real-time fMRI setup and feedback calculation”).

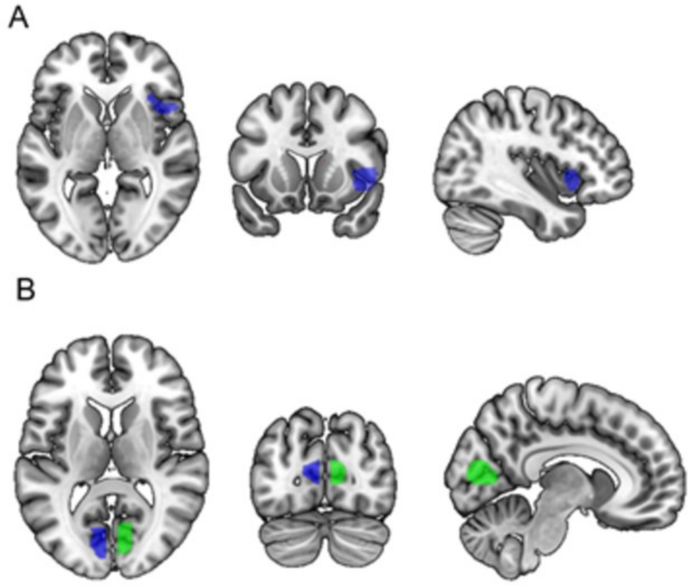

The masks used for time-series extraction during rt-fMRI neurofeedback training were obtained from the Stanford 90ROI functional atlas (fROIs). ROIs in this atlas were created by using independent component analysis (ICA) on the group-level resting-state data from 15 healthy participants. The rAIC mask (A) comprises 319 voxels and the two V1 masks (B) contain 333 voxels, respectively.

Figure S2. RAIC activity over training runs right V1 and left V1 group pooled (related to Figure 3).

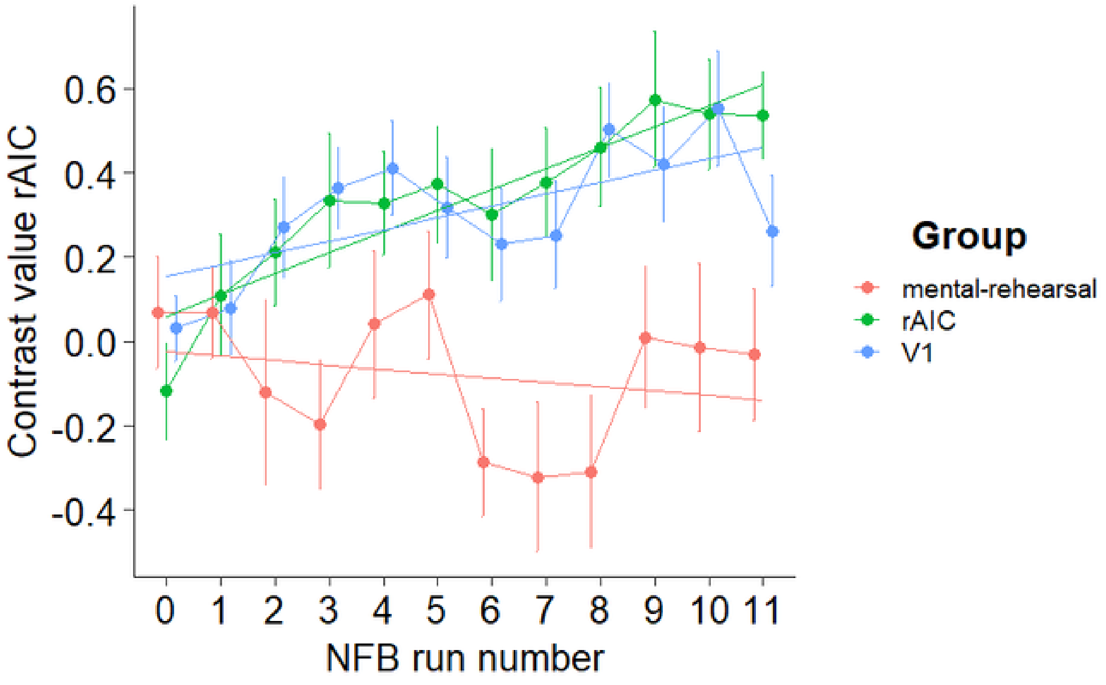

Figure S3. RAIC activity over training runs V1 group separately (related to Figure 3).

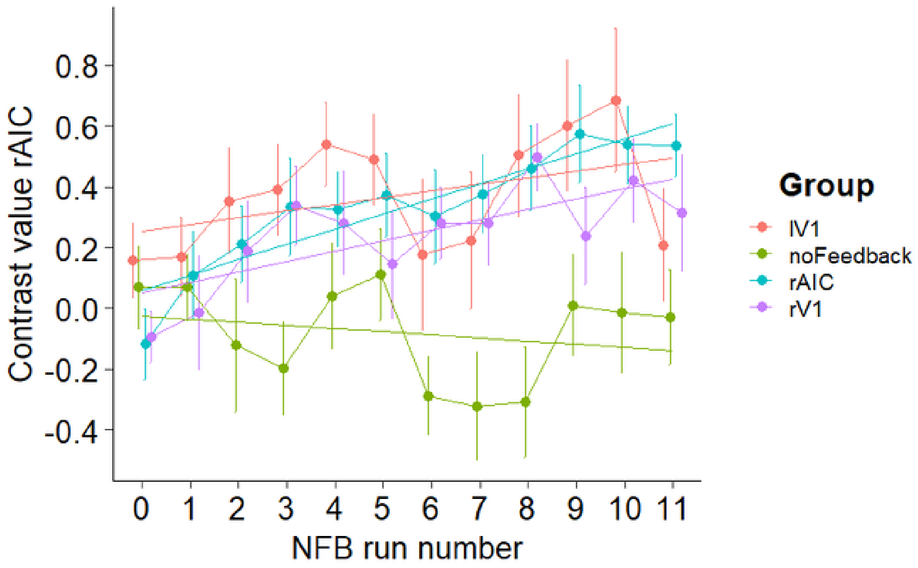

Figure S4 Whole-brain group × session interaction (related to Figure 3).

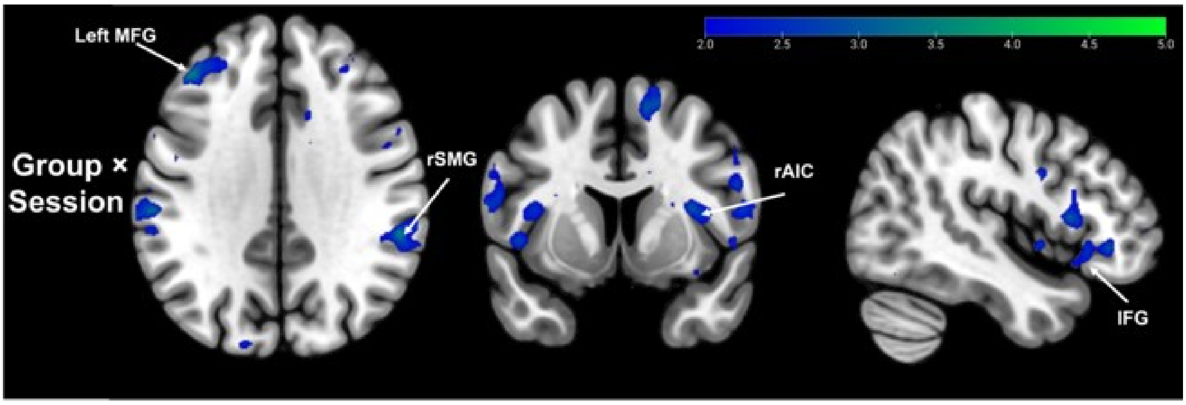

The interaction effect is tresholded at voxel-level $p < 0.005$ (uncorrected). Named brain regions revealed significant activation at voxel-level ($p < 0.001$ uncorrected). Abbreviations: MFG= middle frontal gyrus, rSMG = right supramarginal gyrus, IFG = inferior frontal gyrus

Figure S5. ANT results without outliers (related to Figure 4).

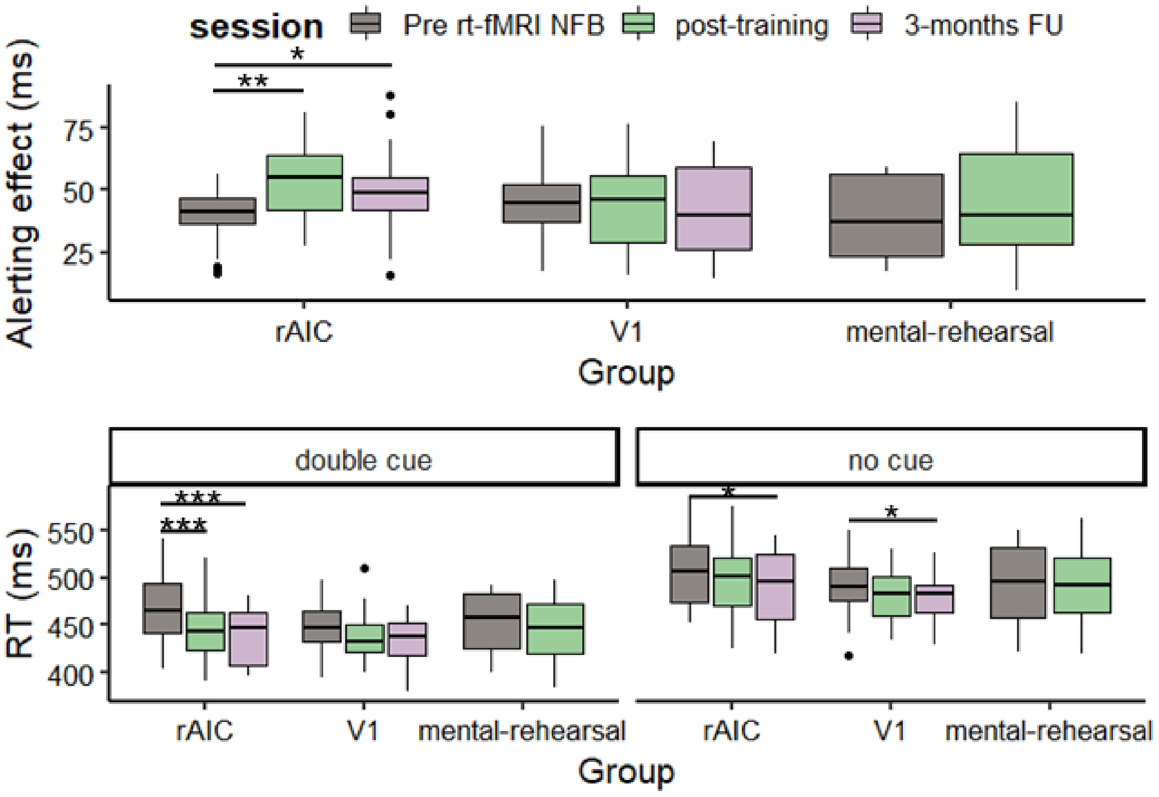

Participants in the rAIC group showed an increased alerting effect right after rt-fMRI neurofeedback training (post-training) compared to before (**$p < 0.01$). This effect was also evident three months later (*$p < 0.05$). The lower panel shows that this effect was driven by faster reaction times for the double cue condition for the examined time intervals. Post-hoc test showed that participants in the rAIC group responded significantly faster to double cue trials immediately after training compared to before rt-fMRI neurofeedback (***$p<0.001$). RTs remained shorter during the three months FU compared to before neurofeedback training (***$p<0.001$). RT for no cue condition decreased from pre rt-fMRI to the 3-months FU session in the rAIC and V1 group (*$p<0.05$).

2. Supplementary tables

Table S1. Mental strategies during baseline run neurofeedback session 02 (related to Figure 3).

rAIC	V1	Mental-rehearsal
Singing song in head	Singing song in head	Recall superbowl position
Imagine job interview	Thinking about boyfriend	Think about last vacation
Singing a song in head	Visualizing self at the beach	Think about first day in University after Covid
Writing paper	Spell words backwards	Think about medical exam
Visualize way to study	Recall friends	Imagine running
Visualize anatomy human body	Mathematical operations	Mathematical operations
Mathematical operations	Scheduling week	Think about thermodynamics
Imagine running in forest	Recall Chinese words	Plan which strategy to use in next runs
Imagine hiking	Singing song in head	Recall university lecture
Planning website creation	Recalling Spanish	Imagine how walk at beach
Mathematical operations	Playing chess in head	
Think about master thesis	Thinking about homework	
Think about thesis	Mathematical operations	
Think about fearful situation	Mathematical operations	
Mathematical operations	Mathematical operations	
Historical facts	Mathematical operations	
Remember bus routes	Spell words backwards	
Recall animal names	Count backwards in steps of six	
Think about own dog	Imagine doing sport	
Imagine herself at beach	Mathematical operations	
Think playing with dog	Imagine a point on screen	
Remember last vacation	Recall a presentation that has to be done	
	Remember thinks to do	

Table S2. Mental strategies during transfer run neurofeedback session 03 (related to Figure 3)

rAIC	V1	Mental-rehearsal
Thinking about a fearful situation	Remember meaning of Chinese signs	Listing countries in alphabetic order
Thinking about universe	Imagine movements	Thinking about vegan snacks
Singing song in head	Spelling words backwards	Imagine snorkeling
Thinking of writing a paper	Thinking about a painful situation	List animal names in three different languages
Playing shooter game	Recalling a way	Thinking about what doing the next day
Mathematical operations	Singing song in head	Recall random numbers and letters
Thinking about philosophical questions	Imagine moving in a room	Thinking about programming
Naming parts of own body	Counting backwards in Chinese (foreign language)	Listing alphabet reverse
Imagine hearing loud music	Fibonacci sequence	Imagine the way to get home
Mathematical operation	Mathematical operations	Imagine walking on grass
Imagine playing volleyball	Imagine walking in a forest	
Mathematical operation	Mathematical operations	
Thinking about travelling	Thinking about friends	
Thinking about things that bother	Thinking about shopping	
Imagine successful situations	Counting binary system	
Fix gaze at neurofeedback stimuli	Imagine playing the piano	
Recalling borders of countries	Envision house-warming party	
Meditation technique	Spelling words backwards	
Naming geographical locations	Thinking about previous work	
Thinking about scary movie	Imagine doing sport	
Thinking about girlfriend	Thinking about ex-partner	
Imagine cooking	Mathematical operations	
Imagine next vacation	Planning study schedule	
	Visualize countries on a map	

Participants reported trying a wide range of mental strategies to increase the feedback signal. Strategies often changed between runs. Even though a detailed classification of the regulation strategies was not conducted, visual inspection of the different strategies suggests that they were similar across groups. In all groups some participants reported to have used mathematical operations (e.g., division, multiplication, or Fibonacci sequence), emotional memories (e.g., thinking of sad, successful, or fearful situations), imagine movement (e.g., move body parts or walk in the forest) or auditory strategies (e.g., think of loud music or sing a song). Importantly, strategies reported during transfer runs were similar to strategies used during the baseline runs.

Table S3. CRED-nf checklist related to STAR Methods

		**CRED-nf best practices checklist 2020**	
**Domain**	**Item #**	**Checklist item**	**Reported on page #**
**Pre-experiment**			
	1a	Pre-register experimental protocol and planned analyses	20
	1b	Justify sample size	n/a
**Control groups**			
	2a	Employ control group(s) or control condition(s)	22
	2b	When leveraging experimental designs where a double-blind is possible, use a double-blind	Single blind
	2c	Blind those who rate the outcomes, and when possible, the statisticians involved	n/a
	2d	Examine to what extent participants and experimenters remain blinded	16
	2e	In clinical efficacy studies, employ a standard-of-care intervention group as a benchmark for improvement	
**Control measures**			
	3a	Collect data on psychosocial factors	12
	3b	Report whether participants were provided with a strategy	8
	3c	Report the strategies participants used	SI Table S1 S2
	3d	Report methods used for online-data processing and artefact correction	24
	3e	Report condition and group effects	
**Feedback specifications**			
	4a	Report how the online-feature extraction was defined	24
	4b	Report and justify the reinforcement schedule	22-24
	4c	Report the feedback modality and content	22
	4d	Collect and report all brain activity variable(s) and/or contrasts used for feedback as displayed to experimental participants	24
	4e	Report the hardware and software used	22-24
**Outcome measures**			
Brain	5a	Report neurofeedback regulation success based on the feedback signal	n/a
	5b	Plot within-session and between-session regulation blocks of feedback variable(s), as well as pre-to-post resting baselines or contrasts	Fig3
	5c	Statistically compare the experimental condition/group to the control condition(s)/group(s) (not only each group to baseline measures)	7-8
**Behavior**	6a	Include measures of clinical or behavioral significance, defined a priori, and describe whether they were reached	9-10
	6b	Run correlational analyses between regulation success and behavioral outcomes	11
**Data storage**			
	7a	Upload all material analysis scripts, code, and raw data used for analyses, as well as final values, to an open access data repository, when feasible	19
